# The Role of Endometriosis in Intestinal Inflammation: A Combined Mendelian Randomization and Cellular Study

**DOI:** 10.1111/jcmm.70799

**Published:** 2025-09-08

**Authors:** Zhigang Li, Fang Wang, Ernv Kang, Xiaoguang Zhen, Jianli Liu, Wenhao Wang

**Affiliations:** ^1^ Department of Clinical Laboratory Second Hospital of Shanxi Medical University Taiyuan Shanxi China; ^2^ Department of Obstetrics and Gynecology Second Hospital of Shanxi Medical University Taiyuan Shanxi China

**Keywords:** endometriosis, inflammation, inflammatory bowel disease, intestinal barrier dysfunction, mendelian randomization

## Abstract

This study aims to assess whether endometriosis causally increases the risk of IBD through Mendelian randomisation (MR) analysis and to elucidate potential mechanisms using in vitro experiments. A two‐sample Mendelian randomisation (MR) analysis was conducted using genome‐wide association study datasets for endometriosis and IBD, including ulcerative colitis and Crohn's disease. Causal inference was assessed using inverse variance weighting, MR‐Egger, and weighted median methods, with MR‐PRESSO used to detect horizontal pleiotropy. Additionally, peritoneal fluid from endometriosis patients (EM‐PF) and healthy controls (CN‐PF) was used to treat Caco‐2 cells. Cell viability, apoptosis, barrier function, and inflammatory cytokine expression were analysed using MTT, TUNEL, transepithelial electrical resistance (TEER), Western blot, and qRT‐PCR assays. MR analysis identified a significant causal association between endometriosis and IBD risk (IVW: *β* = 0.15–0.47, *p* < 0.05). Sensitivity analyses confirmed result robustness with minimal pleiotropy and heterogeneity. Experimental results showed that EM‐PF significantly reduced Caco‐2 cell viability and TEER values while increasing apoptosis and epithelial permeability (*p* < 0.01). Western blot and immunofluorescence staining revealed a marked decrease in tight junction proteins (ZO‐1, Occludin) and an upregulation of inflammatory cytokines (IL‐6, IL‐8, IL‐1β) in the EM‐PF group (*p* < 0.01). Our findings provide genetic and experimental evidence supporting a causal role of endometriosis in increasing IBD risk. Endometriosis‐associated peritoneal fluid may contribute to gut inflammation and epithelial dysfunction, offering new insights into the pathophysiological connection between these conditions.

## 1 Introduction

Endometriosis is a chronic, oestrogen‐dependent, inflammatory disorder characterised by the presence of functional endometrial‐like tissue outside the uterine cavity, predominantly in the pelvic peritoneum, ovaries, and other extrauterine locations. It affects approximately 10% of women of reproductive age and is associated with chronic pelvic pain, dysmenorrhea, and infertility, imposing a substantial burden on both quality of life and healthcare resources [1, 2]. Beyond the reproductive system, endometriosis has systemic implications and is increasingly recognised as a disease with multi‐organ involvement and immune dysregulation [3, 4].

In recent years, a growing body of epidemiological evidence has demonstrated an increased prevalence of comorbidities in women with endometriosis, including autoimmune diseases and chronic inflammatory conditions [5, 6]. Among these, inflammatory bowel disease (IBD), comprising ulcerative colitis (UC) and Crohn's disease (CD), has attracted particular interest. IBD is a chronic, relapsing inflammatory disorder of the gastrointestinal tract, with a complex aetiology involving genetic susceptibility, immune dysregulation, microbial imbalance, and environmental factors [7]. Several large‐scale population‐based studies have reported a bidirectional association between endometriosis and IBD, wherein women with endometriosis exhibit a heightened risk of developing IBD, and vice versa [8, 9]. These findings suggest a potential pathophysiological link between the two diseases.

Despite the well‐documented clinical association, the underlying mechanisms driving the comorbidity between endometriosis and IBD remain poorly understood. Both conditions share common features, including chronic systemic inflammation, elevated pro‐inflammatory cytokine profiles (e.g., IL‐6, IL‐8, TNF‐α), and alterations in immune responses that contribute to disease progression [10, 11]. Furthermore, abnormalities in epithelial barrier function, which play a critical role in maintaining intestinal homeostasis, have been implicated in the pathogenesis of both diseases [12, 13]. However, whether endometriosis causally contributes to the development of IBD, or whether their coexistence is attributable to shared genetic and environmental factors, has yet to be clarified.

Mendelian randomization (MR), an analytical method that uses genetic variants as instrumental variables to assess causal inference, offers a promising approach to disentangle correlation from causation in observational studies [14]. MR analyses can minimise confounding and reverse causality by leveraging genetic data as unconfounded proxies for exposures [15]. To date, limited research has employed MR to investigate the causal relationship between endometriosis and IBD. Moreover, functional experimental validation of the impact of endometriosis on intestinal health is lacking.

In this study, we aimed to investigate the potential causal association between endometriosis and IBD using a two‐sample MR approach based on large‐scale genome‐wide association study (GWAS) datasets. Additionally, to explore the possible biological mechanisms underlying this association, we conducted in vitro experiments using Caco‐2 intestinal epithelial cells exposed to peritoneal fluid from endometriosis patients. We assessed the effects on cell viability, apoptosis, barrier integrity, and inflammatory cytokine expression. By integrating genetic epidemiology with functional validation, this study provides a comprehensive analysis of the relationship between endometriosis and IBD, offering novel insights into their potential pathophysiological connection. Our findings may guide future research on disease mechanisms and preventive strategies for women with endometriosis who are at increased risk of IBD.

## 2 Materials and Methods

### 2.1 Mendelian Randomization

#### 2.1.1 Study Design

This study is based on three core assumptions: (1) Genetic variations should be associated with exposure; (2) Genetic variations should be independent of confounding factors; (3) Genetic variations should only affect the outcome through exposure. We utilised the endometriosis GWAS dataset (finngen_R9_N14_ENDOMETRIOSIS) as the exposure factor. The population selection, gene genotyping, and relevant baseline data involving GWAS data have been previously reported in other studies. Data collection was approved by the original GWAS ethics committee, and this MR study follows the guidelines of the Strengthening the Reporting of Observational Studies in Epidemiology for Mendelian Randomization (STROBE‐MR).

#### 2.1.2 IBD‐Related GWAS Meta‐Analysis

To investigate the relationship between endometriosis and the risk of developing IBD in the UK, we conducted a search for IBD‐related GWAS data. The study included six GWAS datasets related to IBD: benign rectal tumour, finn‐b‐CD2_BENIGN_RECTUM; IBD, ieu‐a‐31; CD, ieu‐a‐12; CD, ieu‐a‐30; UC, ieu‐a‐32; UC, ieu‐a‐973. Among these, finn‐b‐CD2_BENIGN_RECTUM included 2108 cases, 216,684 controls, and 16,380,466 SNP loci; ieu‐a‐31 included 12,882 cases, 21,770 controls, and 12,716,084 SNP loci; ieu‐a‐12 included 17,897 cases, 33,977 controls, and 1,248,880 SNP loci; ieu‐a‐30 included 5956 cases, 14,927 controls, and 12,276,506 SNP loci; ieu‐a‐32 included 6968 cases, 20,464 controls, and 12,255,197 SNP loci; ieu‐a‐973 included 6687 cases, 19,718 controls, and 1,243,971 SNP loci. As previous studies reported shared inflammatory and genetic features of IBD, the benign rectal tumour dataset was included [16, 17].

#### 2.1.3 Mendelian Randomization Estimation

Candidate instrumental variables for the two‐sample MR study were selected from SNPs associated with endometriosis (*p* < 5e‐06). Subsequently, a clumping procedure was applied with *r* ^2^ = 0.01 and a clumping window of 10 Mb to remove linkage disequilibrium variants from the instrumental variables. The selection and quality control of instrumental variables were computed using TwoSampleMR (v 0.5.6) [18]. Finally, causal relationships between endometriosis and IBD occurrence were analysed using inverse variance weighting (IVW), MR‐Egger, and weighted median (WM) methods. IVW estimates the causal effect of genes on the disease through weighted averaging; MR‐Egger estimates the causal effect of genes on the disease by fitting a linear regression model and detects and corrects for genetic bias through Egger regression; WM provides robust estimates in the presence of genetic bias. Additionally, Cochrane's *Q* statistic was used to assess heterogeneity, and outliers were removed if detected, followed by re‐evaluation of MR causal relationships. MR‐PRESSO tested for horizontal pleiotropy and provided corrected estimates. Statistical analysis and data visualisation were performed in R software version 4.1.3.

### 2.2 In Vitro Experiments

#### 2.2.1 Peritoneal Fluid Collection and Preparation

Peritoneal fluid (PF) samples were collected from women undergoing laparoscopic surgery at Second Hospital of Shanxi Medical University, between May 2021 and May 2022. Ethical approval was obtained from the Institutional Ethics Committee of Second Hospital of Shanxi Medical University (Approval Number: (2021) YX No. 135), and informed consent was obtained from all participants prior to enrolment.

Endometriosis‐Associated Peritoneal Fluid (EM‐PF): Peritoneal fluid was collected from women diagnosed with endometriosis by histopathological examination following laparoscopic surgery. Patients included had stage III–IV endometriosis according to the revised American Society for Reproductive Medicine (rASRM) classification system. None of the patients had received hormonal or immunosuppressive therapy in the 3 months preceding surgery. A total of 10 patients were included in the EM‐PF group.

Control Peritoneal Fluid (CN‐PF): Control PF was obtained from women without endometriosis, confirmed by laparoscopy performed for benign gynaecological conditions such as ovarian cysts or tubal ligation. These participants had no clinical or histological evidence of endometriosis, pelvic inflammatory disease, or malignancy. A total of 8 patients were included in the CN‐PF group.

Peritoneal fluid samples were collected under sterile conditions at the beginning of surgery, prior to any manipulation that might disrupt peritoneal structures. Fluids were centrifuged at 1500 × g for 10 min at 4°C to remove cellular debris, aliquoted, and stored at −80°C until further use.

#### 2.2.2 Cell Culture and Grouping

The human colorectal adenocarcinoma cell line, Caco‐2, was obtained from the American Type Culture Collection (ATCC, Manassas, VA, USA). Cells were maintained in Dulbecco's Modified Eagle Medium (DMEM; Gibco, USA), supplemented with 10% fetal bovine serum (FBS; Gibco, USA), 1% penicillin–streptomycin solution (Gibco, USA), and incubated at 37°C in a humidified atmosphere containing 5% CO_2_. Cells were passaged at 80%–90% confluence using 0.25% trypsin–EDTA (Gibco, USA).

Caco‐2 cells were divided into three groups: Control medium group (CM): Cells were cultured in standard complete DMEM without any peritoneal fluid supplementation. CN‐PF group: Cells were cultured in DMEM supplemented with 50% (v/v) CN‐PF, simulating exposure to the peritoneal environment of healthy individuals. EM‐PF group: Cells were cultured in DMEM supplemented with 50% (v/v) EM‐PF, to evaluate the effect of endometriosis‐associated peritoneal fluid on intestinal epithelial cells.

#### 2.2.3 MTT Assay

Cells were seeded in 96‐well plates at 5 × 10^3^ cells per well and cultured until confluence. Cells were subsequently treated according to the designated experimental conditions. At 0, 24, 48, and 72 h, the cell culture plates were taken out, and 20 μL of 5 mg/mL MTT solution (Biyuntian Company, C009S, China) was added to each well for 4 h of incubation. After incubation, the supernatant was carefully removed, and 150 μL of DMSO was added to dissolve the crystals. The mixture was gently shaken at room temperature to mix. The optical density (OD) values of each well were measured at 570 nm using a microplate reader to reflect the changes in cell viability.

#### 2.2.4 TUNEL Assay

The TUNEL assay was performed following the standard procedure of the TUNEL kit. First, treated cells were fixed with 4% paraformaldehyde (Biyuntian Company, P0099‐100 mL, China) for 10 min and washed twice with PBS (Beyotime, BL302A, China). Then, proteinase K (20 μg/mL) was used to incubate the cells at room temperature for 10 min. According to the TUNEL kit instructions (Biyuntian Company, C1086, China), TUNEL reaction solution was added, and the cells were incubated at 37°C for 1 h, allowing the DNA fragmented ends of apoptotic cells to bind with fluorescently labelled dUTP. After incubation, the excess reaction solution was washed off with PBS, followed by DAPI staining for nuclear counterstaining. The apoptotic cells were observed and photographed under a fluorescence microscope. The percentage of apoptotic cells in each field was calculated by randomly selecting five fields and expressed as the percentage of positive cells representing the apoptotic level.

#### 2.2.5 Transepithelial Electrical Resistance (TEER) Measurement

Caco‐2 cells were seeded onto the upper membrane of Transwell chambers. After the cells reached full confluence, different treatment media were added to each group, and the cells were cultured until the designated time points. Prior to TEER measurement, the media were replaced with HBSS buffer (Thermo Fisher, 14065056, USA) to balance the resistance and incubated for 30 min. Subsequently, a commercial TEER meter was used to measure the resistance of each chamber. Electrodes were immersed in both the upper and lower chambers of the Transwell, and the resistance values were recorded for each group. All measurements were performed in a constant‐temperature incubator (37°C, 5% CO_2_). The net resistance of the cell layer (i.e., the total resistance minus the bare membrane resistance) was calculated by measuring the resistance of the bare membrane. The normalised TEER values were then calculated by adjusting for the chamber area.

#### 2.2.6 Immunofluorescence Staining

FITC‐labelled dextran was added to the upper chamber of the Transwell for a 2‐h incubation. Afterward, the amount of FITC‐dextran in the lower chamber fluid was measured. At the same time, the cells in each group were stained with antibodies against ZO‐1 (Abcam, ab276131, UK) and Occludin (Abcam, ab216327, UK) following the standard immunofluorescence staining protocol. Cells were fixed on the Transwell membrane or slides with 4% paraformaldehyde for 10 min and washed twice with PBS. Then, the cells were permeabilised with 0.1% Triton X‐100 (Beyotime, P0096, China) for 10 min. After blocking with 5% bovine serum albumin at room temperature for 30 min, primary antibodies against ZO‐1 or Occludin were added and incubated overnight at 4°C. The next day, after washing with PBS, fluorescence‐labelled secondary antibodies were added and incubated for 1 h at room temperature. Excess antibodies were washed off with PBS. Finally, the nuclei were stained with DAPI, and the distribution of tight junction proteins was observed by fluorescence microscopy. Fluorescence images were captured to record the distribution and intensity of the fluorescence signal in each group.

#### 2.2.7 qRT‐PCR

Total RNA was extracted from the cells using TRIzol reagent (Invitrogen, 15596026, USA). The RNA was then reverse‐transcribed into cDNA using a reverse transcription kit (Thermo Fisher Scientific, K1621, USA) according to the manufacturer's instructions. The qRT‐PCR reaction mix consisted of cDNA template, SYBR Green dye (Biyuntian, D7405, China), specific primers (Table 1), and RNase‐free water, with a total reaction volume of 20 μL. The amplification was performed using a fluorescent quantitative PCR machine (e.g., ABI 7500) with the following programme: 95°C for 2 min for pre‐denaturation, followed by 40 cycles of 95°C for 15 s, 60°C for 30 s, and 72°C for 30 s. Fluorescence signals were collected at the end of each cycle. Relative expression levels of target genes were calculated using the 2^−ΔΔCt^ method, normalised to the expression of the internal reference gene GAPDH.

**TABLE 1:** The primers used in the present study.

	Forward primer (5′ – 3′)	Reverse primer (5′ – 3′)
IL‐6	TGCGATGGAGTCAGAGGAAAC	AAGCTGAAGTCATGCACGAAG
IL‐8	TGTTCCACTGTGCCTTGGTT	TGCTTCCACATGTCCTCACA
IL‐1β	CCAAACCTCTTCGAGGCACA	AGCCATTTCACTGGCGA
GAPDH	TGTGAACGGATTTGGCCGTA	GATGGTGATGGGTTTCCCGT

#### 2.2.8 Western Blotting

Total proteins were extracted from cells using RIPA lysis buffer (Biyuntian, P0013B, China) and incubated on ice for 30 min with gentle shaking. Afterward, the cell lysates were centrifuged at 12,000 g for 10 min at 4°C, and the supernatants were collected. Protein concentrations were measured using the BCA method, and equal amounts of protein were mixed with SDS sample buffer and boiled for 5 min for denaturation. The samples were then loaded onto a 12% SDS‐PAGE gel (Guangzhou Yujia Company, P0014A, China) for electrophoresis. After electrophoresis, the proteins were transferred to a PVDF membrane (transfer conditions: 100 V for 90 min or 300 mA for 60 min). The membrane was blocked with 5% non‐fat milk solution at room temperature for 1 h. Primary antibodies, such as those against Bax (Abcam, ab182733, UK), Bcl‐2 (Abcam, ab182858, UK), Cytochrome c (Abcam, ab133504, UK), ZO‐1 (Abcam, ab276131, UK), Occludin (Abcam, ab216327, UK), Claudin‐3 (Abcam, ab317319, UK), or Claudin‐4 (Abcam, ab52156, UK), were added and incubated overnight at 4°C. After washing, HRP‐labelled secondary antibodies were added and incubated for 1 h. Protein bands were visualised using ECL reagent (Ruishuo Bio, WBKLS0500, China), and relative protein expression levels were quantified using ImageJ software, with β‐actin as the internal reference for normalisation.

### 2.3 Statistical Analysis

Data were analysed using GraphPad Prism 8.0.2. Results are expressed as mean ± SD. Normality was assessed with the Shapiro–Wilk test. For comparisons between two groups, the Student's *t*‐test was used; for multiple group comparisons, one‐way ANOVA with Tukey's post hoc test was applied. A *p*‐value of < 0.05 was considered statistically significant.

## 3 Results

### 3.1 Causal Relationships Between Endometriosis and IBD‐Related Diseases

**FIGURE 1:**
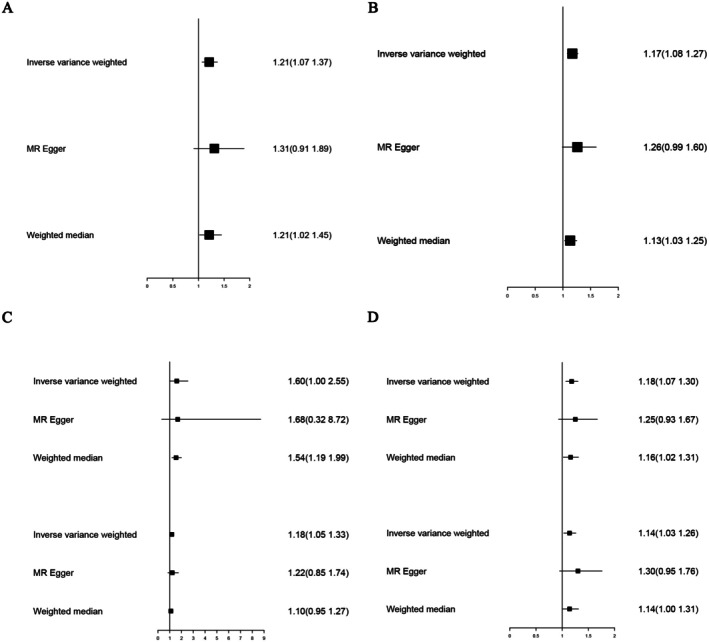
Forest plot depicting the causal relationship between endometriosis and Inflammatory Bowel Disease (IBD) using Inverse Variance Weighting (IVW), MR‐Egger, and Weighted Median (WM) analyses. (A) Association between endometriosis and overall IBD risk. (B) Association between endometriosis and ulcerative colitis (UC). (C) Association between endometriosis and Crohn's disease (CD). (D) Sensitivity analyses for the associations shown in (A–C). Each plot displays the odds ratio (OR) and 95% confidence interval (CI) for the risk of IBD outcomes per genetically predicted increase in endometriosis liability. Squares represent point estimates, and horizontal lines indicate 95% CIs.

We analysed the causal associations between endometriosis and six IBD‐related GWAS datasets (finn‐b‐CD2_BENIGN_RECTUM, ieu‐a‐31, ieu‐a‐12, ieu‐a‐30, ieu‐a‐32, ieu‐a‐973). IVW results showed a causal association between endometriosis and benign rectal tumour (*β* = 0.19, 95% CI: 0.07–0.31, *p* = 0.002), endometriosis and IBD (*β* = 0.15, 95% CI: 0.08–0.24, *p* = 8.02E‐05), endometriosis and CD (*β* = 0.47, 95% CI: 0.002–0.94, *p* = 0.049; *β* = 0.17, 95% CI: 0.05–0.28, *p* = 0.005), and endometriosis and UC (*β* = 0.16, 95% CI: 0.07–0.26, *p* = 0.0009; *β* = 0.13, 95% CI: 0.03–0.23, *p* = 0.009) (Figure 1A–C).

### 3.2 Sensitivity Analysis

We conducted sensitivity analysis using the MR‐Egger and WM methods to assess the robustness of the results in examining the causal relationship between endometriosis and IBD‐related diseases. The WM method yielded results similar to IVW (finn‐b‐CD2_BENIGN_RECTUM, *β* = 0.19, *p* = 0.03; ieu‐a‐31, *β* = 0.13, *p* = 0.01; ieu‐a‐12, *β* = 0.43, *p* = 0.001; ieu‐a‐30, *β* = 0.09, *p* = 0.19; ieu‐a‐32, *β* = 0.15, *p* = 0.03; ieu‐a‐973, *β* = 0.13, *p* = 0.07) (Figure 1D). However, MR‐Egger analysis showed no causal relationship (finn‐b‐CD2_BENIGN_RECTUM, *β* = 0.27, *p* = 0.16; ieu‐a‐31, *β* = 0.23, *p* = 0.07; ieu‐a‐12, *β* = 0.52, *p* = 0.65; ieu‐a‐30, *β* = 0.20, *p* = 0.29; ieu‐a‐32, *β* = 0.22, *p* = 0.15; ieu‐a‐973, *β* = 0.26, *p* = 0.12) (Figure 1D), but the trend was consistent with IVW and WM. MR‐PRESSO results indicated no horizontal pleiotropy between exposure and outcome (Table 2) (finn‐b‐CD2_BENIGN_RECTUM, *p* = 0.49; ieu‐a‐31, *p* = 0.18; ieu‐a‐30, *p* = 0.07; ieu‐a‐32, *p* = 0.23; ieu‐a‐973, *p* = 0.73).

**TABLE 2:** Level pleiotropy test for endometriosis and inflammatory bowel disease (IBD).

Database	MR‐PRESSO	*p*
finn‐b‐BENIGN_RECTUM	27.87	0.49
ieu‐a‐31	35.30	0.18
ieu‐a‐12	NA	NA
ieu‐a‐30	40.30	0.07
ieu‐a‐32	33.56	0.23
ieu‐a‐973	16.42	0.73

### 3.3 Validation Analysis

**FIGURE 2:**
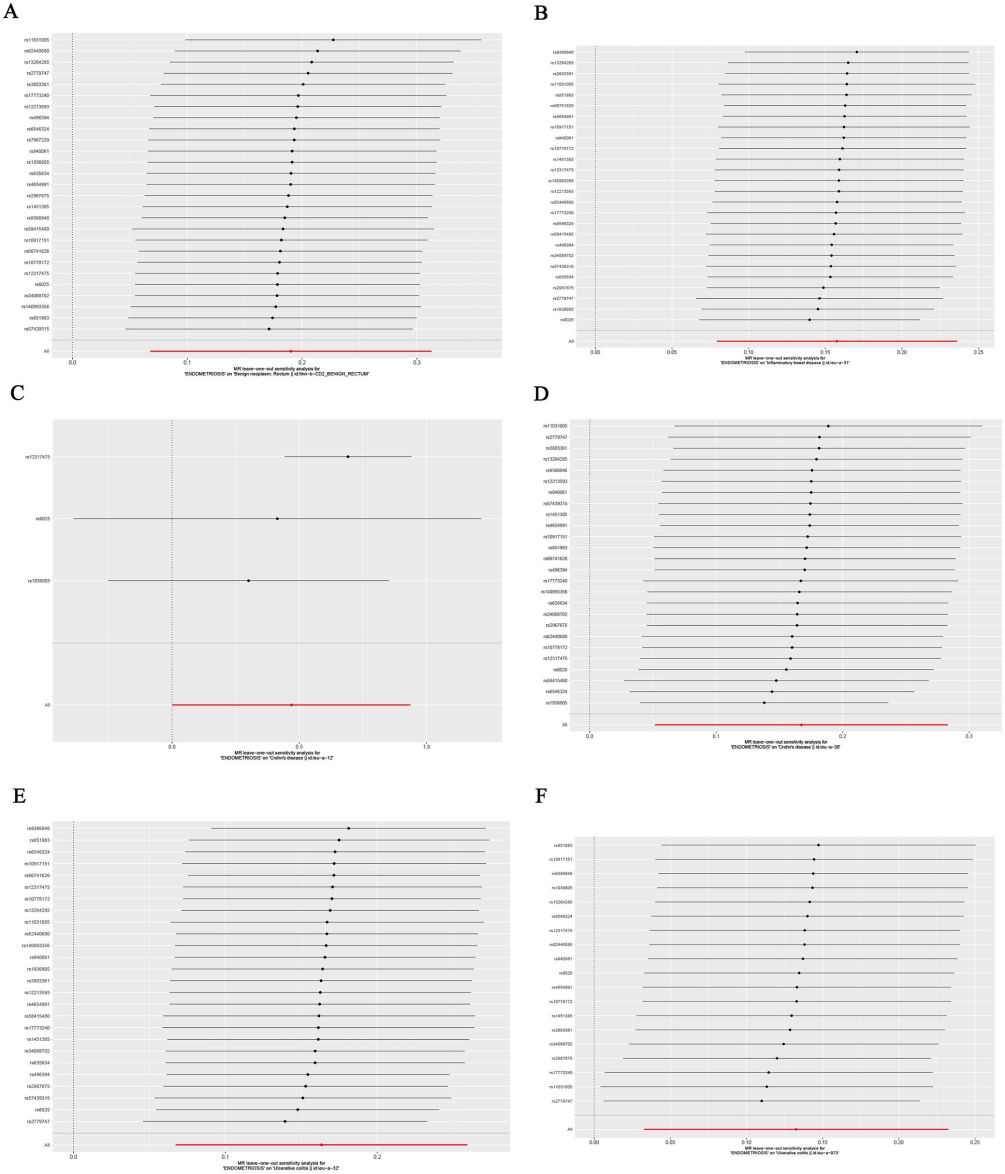
Leave‐one‐out analyses assessing the robustness of the Mendelian randomisation (MR) results for the association between endometriosis and inflammatory bowel disease (IBD) outcomes. (A, B) Leave‐one‐out analysis for endometriosis and IBD (A, finn‐b‐CD2_BENIGN_RECTUM dataset; B, ieu‐a‐31 dataset). (C, D) Leave‐one‐out analysis for endometriosis and Crohn's disease (C, ieu‐a‐12 dataset; D, ieu‐a‐30 dataset). (E, F) Leave‐one‐out analysis for endometriosis and ulcerative colitis (E, ieu‐a‐32 dataset; F, ieu‐a‐973 dataset). Each point represents the inverse variance weighted (IVW) causal estimate after excluding one SNP, with horizontal lines indicating the corresponding 95% confidence intervals.

**FIGURE 3:**
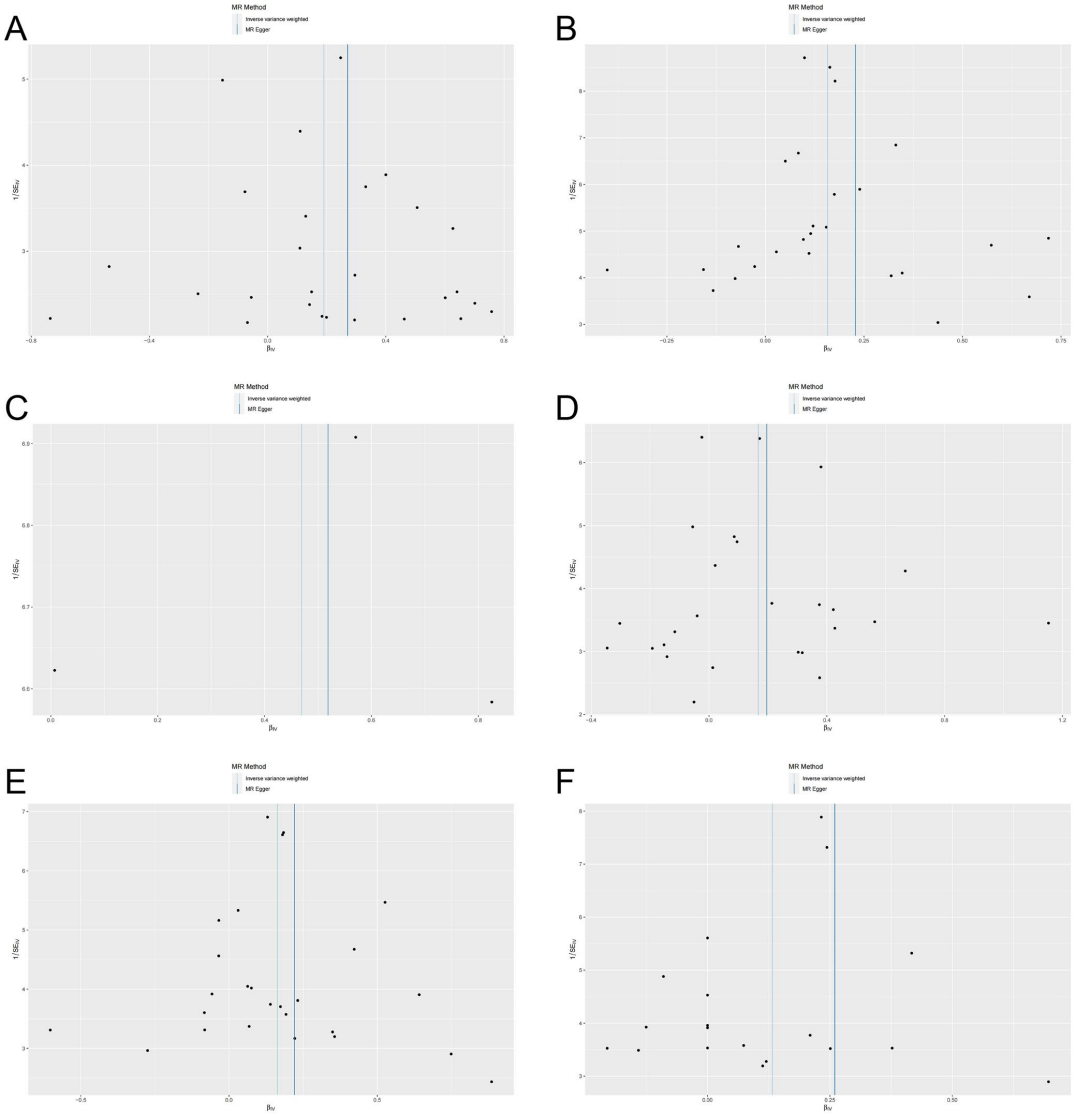
Funnel plot assessing the presence of directional (horizontal) pleiotropy in the Mendelian randomisation (MR) analyses of genetically predicted endometriosis and the risk of inflammatory bowel disease (IBD), including ulcerative colitis (UC) and Crohn's disease (CD). (A, B) Funnel plot for endometriosis and IBD (A, finn‐b‐CD2_BENIGN_RECTUM dataset; B, ieu‐a‐31 dataset). (C, D) Funnel plot for endometriosis and Crohn's disease (C, ieu‐a‐12 dataset; D, ieu‐a‐30 dataset). (E, F) Funnel plot for endometriosis and ulcerative colitis (E, ieu‐a‐32 dataset; F, ieu‐a‐973 dataset). Each point represents a single nucleotide polymorphism (SNP), plotted according to its effect size on the exposure (endometriosis) and outcome (IBD, UC, or CD). The vertical lines indicate the causal estimates from two MR methods: Inverse variance weighted (IVW; blue line) and MR‐Egger regression (grey line).

Heterogeneity refers to the variation in causal estimates obtained from each SNP. Lower heterogeneity indicates more reliable estimates using MR. Cochran's *Q* test showed low heterogeneity for IVW (finn‐b‐CD2_BENIGN_RECTUM, *p* = 0.48; ieu‐a‐31, *p* = 0.21; ieu‐a‐12, 4.64E‐04; ieu‐a‐30, *p* = 0.07; ieu‐a‐32, *p* = 0.27; ieu‐a‐973, *p* = 0.76) and MR‐Egger (finn‐b‐CD2_BENIGN_RECTUM, *p* = 0.43; ieu‐a‐31, *p* = 0.18; ieu‐a‐12, 9.24E‐05; ieu‐a‐30, *p* = 0.06; ieu‐a‐32, *p* = 0.23; ieu‐a‐973, *p* = 0.75) analyses, indicating reliability (Table 3). After individual SNP removal, the association between endometriosis and IBD was not influenced by any single SNP, supporting the robustness of the findings (Figure 2). Furthermore, funnel plots did not show evidence of asymmetry for any SNP (Figure 3).

**TABLE 3:** Cochran's *Q* test for assessing data heterogeneity.

Database	*Q*	*p*
finn‐b‐BENIGN_RECTUM		
MR Egger	25.54	0.43
Inverse variance weighted	25.76	0.48
ieu‐a‐31		
MR Egger	30.00	0.18
Inverse variance weighted	30.47	0.21
ieu‐a‐12		
MR Egger	15.28	9.24E‐05
Inverse variance weighted	15.35	4.65E‐04
ieu‐a‐30		
MR Egger	35.83	0.06
Inverse variance weighted	35.87	0.07
ieu‐a‐32		
MR Egger	28.73	0.23
Inverse variance weighted	28.93	0.27
ieu‐a‐973		
MR Egger	12.77	0.75
Inverse variance weighted	13.50	0.76

### 3.4 Peritoneal Fluid From Endometriosis Patients Inhibits Caco‐2 Cell Viability and Promotes Cell Apoptosis

**FIGURE 4:**
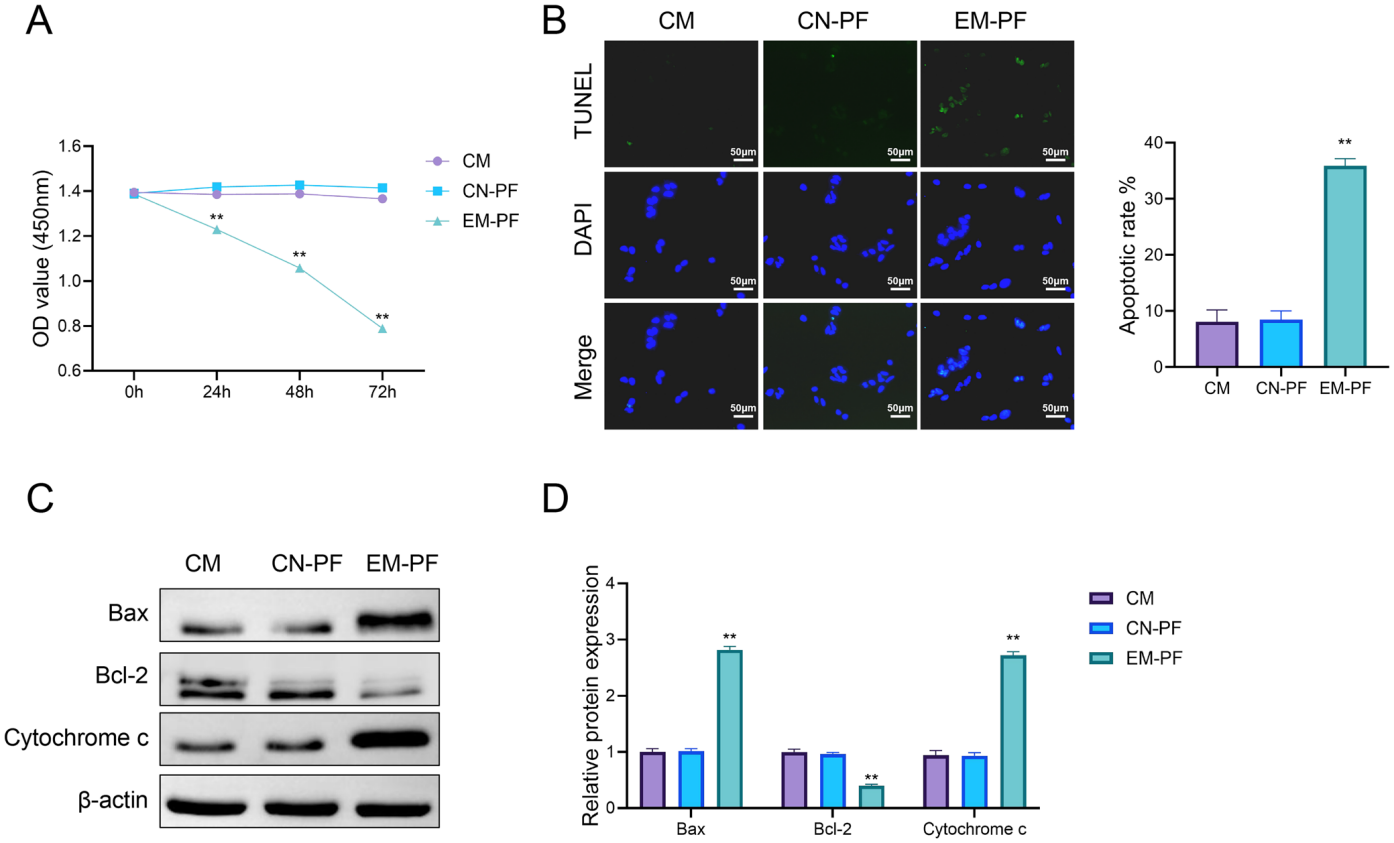
Effect of different treatments on cell apoptosis and protein expression. (A) Cell viability was assessed by OD value at 0, 24, 48, and 72 h. (B) TUNEL staining (green) was used to detect apoptotic cells, with DAPI staining (blue) for nuclei. (C) Western blot analysis of Bax, Bcl‐2, and cytochrome c protein expression in different treatment groups, with β‐Actin as the internal control. (D) Quantification of Bax, Bcl‐2, and cytochrome c protein expression levels based on Western blot results. **p < 0.01 vs. CN‐PF.

To further elucidate the effects of endometriosis‐associated peritoneal fluid on intestinal epithelial cells, we evaluated Caco‐2 cell viability and apoptosis following exposure to EM‐PF. MTT assay results (Figure 4A) showed that the cell viability of the EM‐PF group was significantly lower than that of the CM and CN‐PF groups, with a time‐dependent difference. Over time, the cell viability in the EM‐PF group further decreased (*p* < 0.01). TUNEL staining was used to detect cell apoptosis, and the results indicated that the apoptosis rate in the EM‐PF group was significantly higher than that in the CM and CN‐PF groups, approximately 40% (*p* < 0.01) (Figure 4B). Western blot analysis (Figure 4C,D) showed that the expression of apoptosis‐related proteins Bax and Cytochrome c was significantly increased in the EM‐PF group (*p* < 0.01), while the expression of the anti‐apoptotic protein Bcl‐2 was significantly reduced (*p* < 0.01). In contrast, no significant differences were observed in the expression of these proteins between the CM and CN‐PF groups. These findings suggest that peritoneal fluid from endometriosis patients may negatively affect intestinal epithelial cell viability and promote apoptosis under in vitro conditions.

### 3.5 Peritoneal Fluid From Endometriosis Patients Inhibits Caco‐2 Cell Barrier Function and Increases Cell Permeability

**FIGURE 5:**
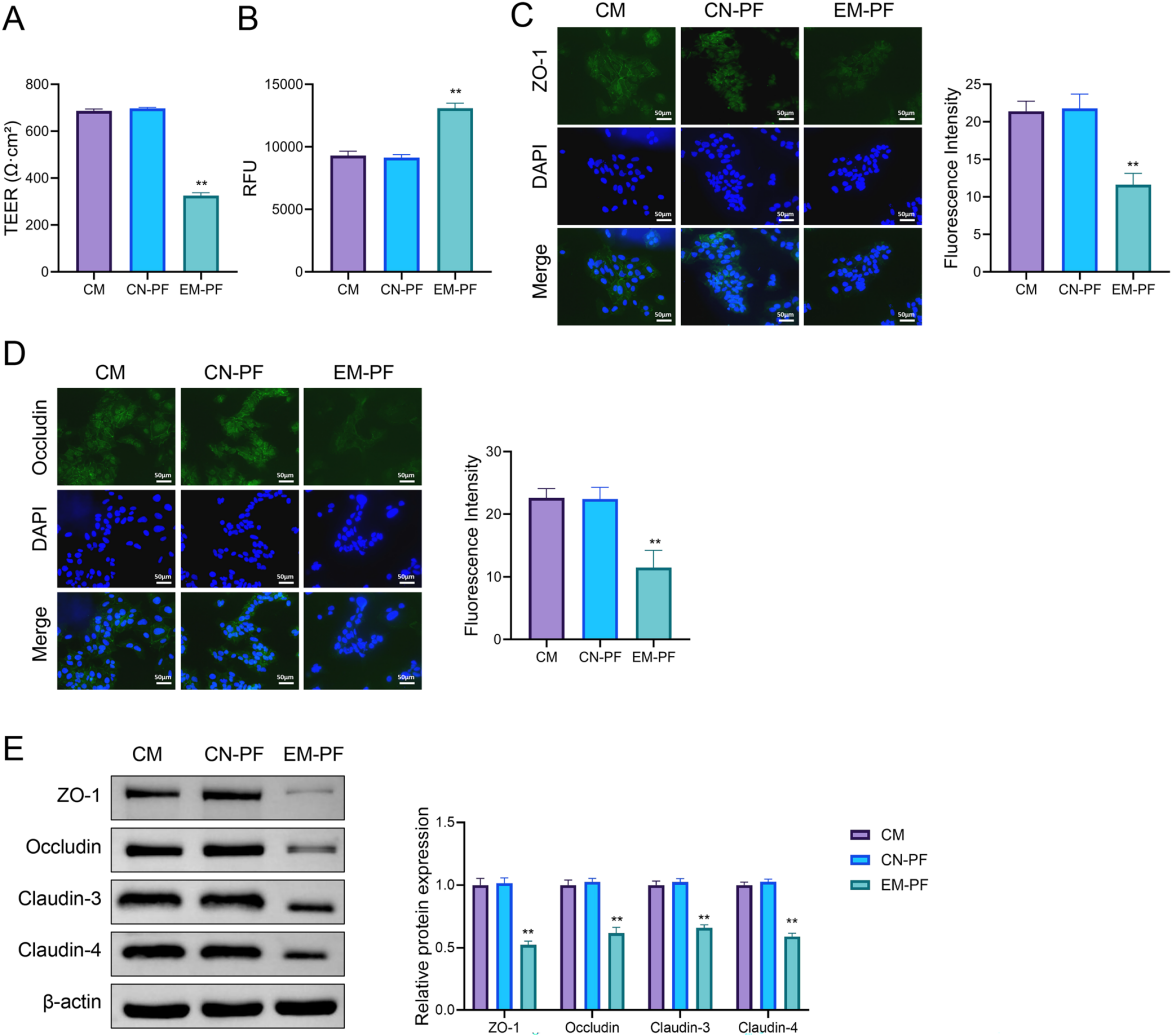
Effect of different treatments on tight junction protein expression and barrier integrity. (A) Cell monolayer barrier integrity was assessed by measuring transepithelial electrical resistance (TEER). (B) Barrier permeability was evaluated by measuring the fluorescence intensity (RFU) in the FITC‐dextran permeability assay. (C) ZO‐1 expression was assessed by immunofluorescence staining (green), with DAPI staining (blue) for cell nuclei. (D) Occludin expression was analysed by immunofluorescence staining (green), with DAPI staining (blue) for cell nuclei. (E) Western blot analysis of ZO‐1, occludin, claudin‐3, and claudin‐4 protein expression in different treatment groups, with β‐Actin as the internal control. Relative protein expression levels were quantified. **p < 0.01 vs. CN‐PF.

Further, we assessed the impact of peritoneal fluid from endometriosis patients on Caco‐2 cell barrier function and cell permeability. TEER measurement results (Figure 5A) indicated that the TEER value in the EM‐PF group was significantly lower than that in the CM and CN‐PF groups (*p* < 0.01). Cell permeability was assessed by measuring the fluorescence intensity of FITC‐dextran penetration (Figure 5B), and the results showed a significant increase in permeability in the EM‐PF group (*p* < 0.01). Immunofluorescence staining results (Figure 5C,D) demonstrated a significant decrease in the fluorescence intensity of ZO‐1 and Occludin in the EM‐PF group compared to the CM and CN‐PF groups (*p* < 0.01). Western blot results (Figure 5E) also revealed a significant reduction in the protein expression of ZO‐1, Occludin, Claudin‐3, and Claudin‐4 in the EM‐PF group (*p* < 0.01). Our results indicate that exposure to endometriosis‐associated peritoneal fluid may compromise epithelial barrier integrity and increase cellular permeability.

### 3.6 Peritoneal Fluid From Endometriosis Patients Promotes IL‐6, IL‐8, and IL‐1β Expression in Caco‐2 Cells

**FIGURE 6:**
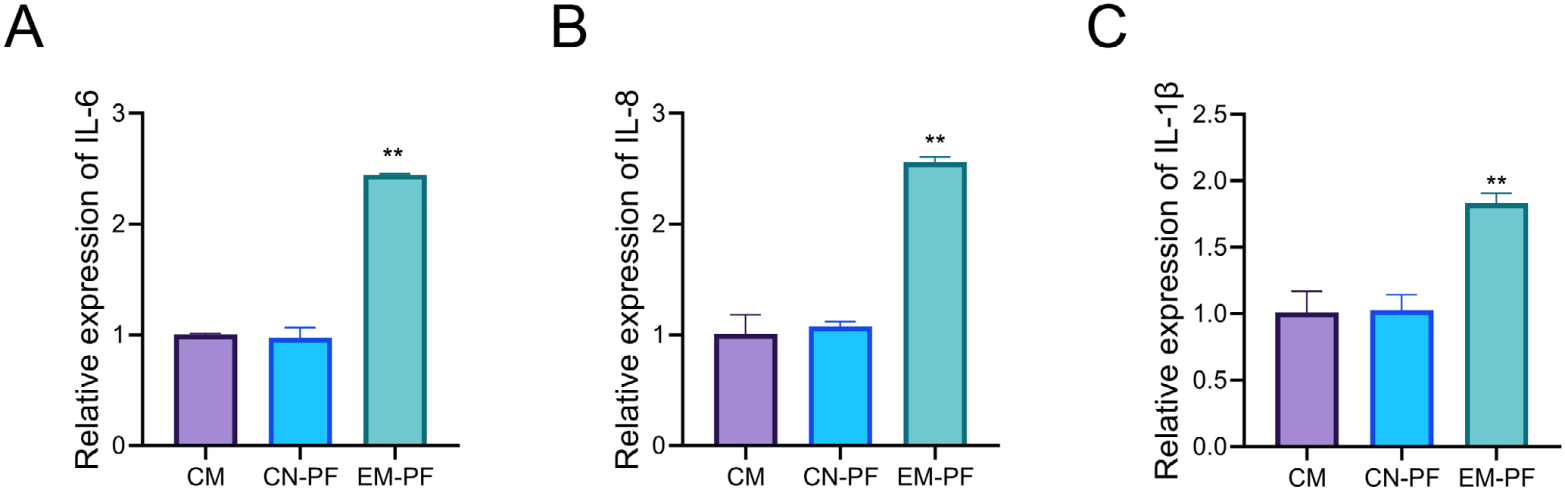
Effect of different treatments on the expression of inflammatory cytokines. Relative expression of (A) IL‐6, (B) IL‐8, (C) IL‐1β in the CM, CN‐PF, and EM‐PF groups, assessed by qRT‐PCR. **p < 0.01 vs. CN‐PF.

Finally, we evaluated the effect of peritoneal fluid on the expression of inflammatory factors in Caco‐2 cells in patients with endometriosis. qRT‐PCR results showed that, compared to the CM and CN‐PF groups, the relative expression levels of IL‐6 (Figure 6A), IL‐8 (Figure 6B), and IL‐1β (Figure 6C) were significantly increased in the EM‐PF group (*p* < 0.01). These data demonstrate that endometriosis‐related peritoneal fluid may contribute to the upregulation of pro‐inflammatory cytokines in intestinal epithelial cells.

## 4 Discussion

In this study, we explored the potential association between endometriosis and IBD by integrating MR analysis with functional in vitro experiments. The MR analysis identified a statistically significant association between genetically predicted endometriosis and an increased risk of IBD, including both UC and CD. Complementary in vitro studies demonstrated that EM‐PF reduced intestinal epithelial cell viability, promoted apoptosis, impaired barrier integrity, and increased the expression of pro‐inflammatory cytokines in Caco‐2 cells. These findings provide genetic evidence and cellular‐level observations that support a potential link between endometriosis and intestinal inflammation. From a clinical perspective, these results suggest that women with endometriosis may be at a higher risk of developing intestinal barrier dysfunction and inflammation, underscoring the importance of considering gastrointestinal health in the comprehensive management of endometriosis patients.

Several population‐based studies have previously reported a higher prevalence of IBD in women diagnosed with endometriosis. For example, Jess et al. [19] conducted a large Danish cohort study and found that women with endometriosis have a significantly increased risk of developing IBD, CD, and UC. Similarly, Lee et al. [20] demonstrated that the coexistence of endometriosis and IBD was associated with greater disease severity and more complicated clinical management. While these observational studies provide valuable epidemiological evidence, they are inherently limited by confounding factors and potential reverse causation. Our use of two‐sample MR, leveraging genetic instruments from large‐scale GWAS, mitigates these limitations by reducing confounding and strengthening causal inference. Notably, our MR analysis suggests a potential causal effect of endometriosis on IBD risk, aligning with prior epidemiological observations and expanding the evidence base by addressing causality.

Furthermore, the in vitro findings from this study are consistent with previous research demonstrating the pro‐inflammatory nature of the peritoneal environment in women with endometriosis. Prior studies have shown that peritoneal fluid from endometriosis patients contains elevated levels of pro‐inflammatory cytokines, such as IL‐6, IL‐8, and TNF‐α, as well as increased oxidative stress markers [21, 22]. These inflammatory mediators have been implicated in disrupting the integrity of the intestinal epithelial barrier, contributing to increased permeability and mucosal inflammation [23]. Our experiments with Caco‐2 cells corroborated these findings, showing that EM‐PF exposure markedly reduced TEER, elevated permeability, and suppressed the expression of tight junction proteins, including ZO‐1 and Occludin. These findings suggest that factors present in the peritoneal fluid of endometriosis patients may directly contribute to intestinal barrier dysfunction, supporting a possible biological connection between endometriosis and IBD.

Importantly, our study highlights the role of apoptosis in mediating intestinal epithelial injury in the context of endometriosis. Increased apoptosis rates, along with elevated expression of pro‐apoptotic proteins Bax and Cytochrome c and reduced expression of the anti‐apoptotic protein Bcl‐2, were observed in Caco‐2 cells treated with EM‐PF. These findings are consistent with previous studies demonstrating increased apoptosis in the intestinal epithelium of IBD patients, which contributes to barrier disruption and disease progression [24]. Thus, our data further support the hypothesis that endometriosis may promote an intestinal microenvironment conducive to inflammation and mucosal damage.

From a pathophysiological perspective, our study suggests that endometriosis and IBD may share common features, as indicated by our genetic and cellular findings. Both diseases exhibit chronic inflammation, immune dysregulation, and epithelial barrier dysfunction as core pathological features [9, 25]. Endometriosis has also been increasingly recognised as a disease with systemic manifestations, involving alterations in immune cell populations, increased production of inflammatory cytokines, and disrupted neuroimmune interactions [10, 26]. Similarly, IBD pathogenesis involves aberrant immune responses to intestinal microbiota, perpetuated by cytokine release and epithelial injury [27]. The overlap in inflammatory pathways, including those mediated by IL‐6 and TNF‐α, may underlie the observed comorbidity between these two conditions.

This overlap in inflammatory and immune dysregulation raises the possibility that specific intracellular signalling pathways may mediate these effects. For example, NF‐κB signalling, a central regulator of inflammatory gene expression, is known to be activated by cytokines such as IL‐6 and TNF‐α and may drive transcriptional programmes leading to epithelial barrier disruption and enhanced intestinal permeability [23]. Similarly, the JAK/STAT pathway has been implicated in cytokine‐induced immune cell activation and chronic mucosal inflammation in IBD [27]. In addition, MAPK/ERK signalling may contribute to epithelial cell apoptosis and tight junction protein dysregulation under inflammatory stress [24]. Together, these pathways could plausibly explain how endometriosis‐associated peritoneal fluid promotes intestinal inflammation and barrier dysfunction, as observed in our experiments. However, it is important to emphasise that these mechanistic insights are hypothetical and remain to be confirmed in future in vivo and in vitro studies.

The clinical implications of our findings are noteworthy. Given the increased risk of gastrointestinal symptoms and potential comorbidity with IBD, clinicians should maintain a high index of suspicion for gastrointestinal involvement in women with endometriosis, particularly in those presenting with chronic abdominal pain, altered bowel habits, or other gastrointestinal complaints. Early identification and interdisciplinary management may help mitigate the impact of coexisting conditions on patients' quality of life.

Despite these important findings, our study has several limitations. First, although MR analysis reduces confounding and reverse causality, the strength of causal inference is still dependent on the validity of the genetic instruments used. While we applied rigorous selection criteria for instrumental variables and performed sensitivity analyses (including MR‐Egger and MR‐PRESSO) to minimise horizontal pleiotropy, unrecognised pleiotropic effects cannot be entirely excluded. Second, the GWAS datasets employed in this study predominantly included participants of European ancestry, limiting the generalisability of our findings to other populations. Future studies incorporating more diverse cohorts are warranted to validate these results. In addition, our study did not include direct clinical cohort data or patient‐level evidence to confirm the association between endometriosis and intestinal barrier dysfunction. Future studies involving well‐designed clinical cohorts are necessary to validate and extend our findings. Finally, the in vitro experiments used Caco‐2 cells as a model of intestinal epithelial cells. Although Caco‐2 cells are commonly used in gut barrier research, they may not entirely reflect the complexity of the in vivo intestinal epithelium, including interactions with immune cells and the gut microbiota.

## 5 Conclusion

In conclusion, this study provides genetic and functional evidence supporting a potential link between endometriosis and IBD. Our findings highlight the possibility that endometriosis‐associated factors may contribute to intestinal barrier dysfunction and inflammation, offering new insights into the pathophysiological connection between these two chronic inflammatory diseases. Future research should focus on elucidating the specific molecular components within endometriosis‐associated peritoneal fluid that mediate these effects and investigate potential therapeutic strategies aimed at preserving gut barrier integrity in affected individuals.

## Author Contributions

**Zhigang Li:** conceptualization (equal), methodology (equal), writing – original draft (equal), writing – review and editing (equal). **Fang Wang:** formal analysis (equal), writing – review and editing (equal). **Ernv Kang:** investigation (equal), supervision (equal). **Xiaoguang Zhen:** software (equal), visualization (equal). **Jianli Liu:** conceptualization (equal), validation (equal). **Wenhao Wang:** data curation (equal), project administration (equal).

## Ethics Statement

Ethical approval was obtained from the Institutional Ethics Committee of Second Hospital of Shanxi Medical University (approval number: (2021) YX No. 135) and conducted in accordance with the Declaration of Helsinki (as revised in 2013).

## Consent

Informed consent was obtained from all participants prior to enrolment.

## Conflicts of Interest

The authors declare no conflicts of interest.

## Data Availability

The data used to support the findings of this study are available from the corresponding author upon request.
